# Dopamine D3 Receptor: Contemporary Views of Its Function and Pharmacology for Neuropsychiatric Diseases

**DOI:** 10.3390/biom11050713

**Published:** 2021-05-11

**Authors:** Philippe De Deurwaerdère, Abdeslam Chagraoui

**Affiliations:** 1 Unité Mixte de Recherche (UMR) 5287, Centre National de la Recherche Scientifique (CNRS), CEDEX, 33000 Bordeaux, France; 2 Neuronal and Neuroendocrine Differentiation and Communication Laboratory, Institute for Research and Innovation in Biomedicine of Normandy (IRIB), Normandie University, UNIROUEN, INSERM, U1239, CHU Rouen, 76000 Rouen, France; 3 Department of Medical Biochemistry, Rouen University Hospital, 76000 Rouen, France

*Biomolecules* has launched a Special Issue entitled “Dopamine D3 Receptor: Contemporary Views of Its Function and Pharmacology for Neuropsychiatric Diseases.” Since its discovery in 1990 by the group of Jean-Charles Schwartz [1], numerous studies have emphasized its brain location, its molecular partners and, more generally, its important roles in central dopaminergic transmission. Nowadays, D3Rs are strongly considered in the treatment of various central nervous system conditions, including schizophrenia, depression, addiction, Parkinson’s disease, and Alzheimer’s disease, to name a few. Numerous drugs acting at D3Rs have been released; some of them are selective, whereas others favor a multitarget approach [2]. The field is progressing fast and in several directions. We have collected research and review articles highlighting interests to target D3Rs in neurological and neuropsychiatric conditions.

The notion of multitarget target drugs is intimately linked with the D3Rs as the newer antipsychotic drugs, including cariprazine [3] display a high affinity for D3Rs. Bela Kiss et al. developed a fundamental approach to the knowledge accumulated on D3Rs [4]. They reviewed the distribution of D3Rs in the central nervous system (CNS) and periphery and its signaling and molecular properties. They also reported the status of ligands available for D3R research (agonists, antagonists and partial agonists), the functional aspects of D3Rs in terms of behavioral impact, and their modulatory role in some neural networks. Partial agonists at D3Rs also have a strong interest in the treatment of cocaine addiction. Powell et al. proposed pharmacological characterization of the selective D3R partial agonist MC-25-41 against cocaine addiction [5]. They confirmed using this specific compound that D3R partial agonism opposes cocaine consumption in rats in a progressive fixed-ratio schedule or in a variable interval of multiple schedules. They also revealed using economic behavioral analysis that MC-25-41 reduces cocaine consumption as the price of the demand increases. MC-25-41, being a long-lasting drug, might be considered a potential drug to test in the clinic. Beyond cocaine addiction, Ruzilawati et al. looked at gene polymorphisms in a Malay male cohort of tobacco smokers [6]. In addition to the D1R and D2R subtypes, they reported an association of gene polymorphism rs7653787 of D3R in smoking behavior.

Numerous dopaminergic agonists have spectacular properties, such as the D2R/D3R agonist quinpirole. Brozka et al. used repeated administration of this agonist to produce an obsessive–compulsive disorder-like behavior in rats [7] as classically described in the literature. Once the abnormal behavior was characterized, they looked at the expression of immediate early genes *Arc* and *Homer1a* in the brain using cellular compartment analysis of temporal activity by fluorescence in situ hybridization. The main change that they reported was in the CA1 region of the hippocampus, whereas the number of cells expressing mRNA for these early genes was not altered by the chronic treatment with quinpirole in frontocortical areas or the nucleus accumbens. The specific contribution of D3 receptors to these effects remains to be elucidated.

There is ongoing progress in the chemistry of D3Rs to produce drugs with a specific pharmacological profile. Using the previous D3R agonist PF592,379 as a primary pharmacophore and a linker to D2R agents, Adhikari et al. produced bitopic compounds that they characterized using BRET-based functional assays. They reported that the chirality of the primary pharmacophore was key to conferring improved D3R potency, selectivity, and G protein signaling bias. The development of pharmacological compounds is also required to develop new probes for imaging D3Rs in the living brain. Hsieh et al. focus on fallypride and fluortriopride, which are used as fallypride (^18^F) and fluortriopride(^18^F) in PET studies [8]. However, their ability to interact with synaptic dopamine is different. The authors reported therein using docking experiments in silico that the ligands differed in terms of binding in the orthosteric site and, using the β-arrestin assay in vitro, that the two compounds act differently on the activity of D3Rs.

In conclusion, the research in the field of D3Rs is very active and covers a broad spectrum of neurobiological functions and neuropsychiatric diseases.
